# Does listening to action-related sentences modulate the activity of the motor system? Replication of a combined TMS and behavioral study

**DOI:** 10.3389/fpsyg.2014.01511

**Published:** 2015-01-05

**Authors:** Claudia Gianelli, Riccardo Dalla Volta

**Affiliations:** ^1^Division of Cognitive Sciences, University of PotsdamPotsdam, Germany; ^2^Department of Medical and Surgical Sciences, Università Magna GraeciaCatanzaro, Italy

**Keywords:** action language, motor system, TMS, motor resonance, interference, replication

## Abstract

The neurophysiological and behavioral correlates of action-related language processing have been debated for long time. A precursor in this field was the study by Buccino et al. (2005) combining transcranial magnetic stimulation (TMS) and behavioral measures (reaction times, RTs) to study the effect of listening to hand- and foot-related sentences. In the TMS experiment, the authors showed a decrease of motor evoked potentials (MEPs) recorded from hand muscles when processing hand-related verbs as compared to foot-related verbs. Similarly, MEPs recorded from leg muscles decreased when participants processed foot-related as compared to hand-related verbs. In the behavioral experiment, using the same stimuli and a semantic decision task the authors found slower RTs when the participants used the body effector (hand or foot) involved in the actual execution of the action expressed by the presented verb to give their motor responses. These findings were interpreted as an interference effect due to a simultaneous involvement of the motor system in both a language and a motor task. Our replication aimed to enlarge the sample size and replicate the findings with higher statistical power. The TMS experiment showed a significant modulation of hand MEPs, but in the sense of a motor facilitation when processing hand-related verbs. On the contrary, the behavioral experiment did not show significant results. The results are discussed within the general debate on the time-course of the modulation of motor cortex during implicit and explicit language processing and in relation to the studies on action observation/understanding.

## Introduction

The target of this replication study is a seminal paper in the field of embodied language processing published by Buccino et al. (2005) that combined TMS and behavioral techniques. In this study the authors found that processing acoustically presented action sentences produced an interference effect as revealed by both neurophysiological and behavioral measures. In the TMS experiment, the authors showed that, when applying single-pulse TMS over the hand primary motor cortex (M1), MEPs amplitude recorded from hand muscles decreased when processing hand-related verbs as compared to foot-related verbs. Similarly, MEPs recorded from leg muscles decreased when processing foot-related verbs as compared to hand-related ones. In the behavioral experiment, using the same stimuli and a semantic decision task, the authors showed a similar interference effect, namely slower RTs when the participants' body effector used to give the motor response corresponded to that involved in the execution of the action expressed by the presented verb. The authors claimed that a specific modulation of hand and foot/leg motor representations is crucially involved in processing language related to the corresponding effectors. These results were further confirmed by other studies (Boulenger et al., 2006; Sato et al., 2008; Dalla Volta et al., 2009; Gough et al., 2013; Marino et al., 2013).

These results raise at least two main theoretical issues. First, they may appear “unexpected” when compared to those obtained when assessing the modulation of the motor system during action observation. In fact, during action observation the motor system is activated, as evidenced by the increase of MEP amplitude recorded from those muscles involved in the execution of the observed action (Fadiga et al., 1995; Strafella and Paus, 2000; Gangitano et al., 2001). Therefore, this apparently opposite direction of the modulation of the motor system, on the one hand, raises the question of whether the same or different motor representations are involved in both action observation and language processing and, on the other, whether the motor system plays a causative role in language processing as it is claimed for the understanding of observed actions.

Second, the modulation pattern of the motor system during language processing is still quite debated. EEG and MEG results support an early recruitment of the motor system during language processing (for review see Pulvermüller et al., 2009). This recruitment has been generally interpreted as a facilitation effect overlapping the one found during action observation. Beside the polarity issue, however, it is worth noting that both the findings of Buccino et al. (2005) and those showing activation in EEG and MEG studies reinforce the notion that the motor system is crucially involved in language processing, by showing an early modulation of neurophysiological measures indexing motor system activity. In line with this assumption, one brain imaging study has shown an overlap within the premotor sectors recruited by the observation of an action and the processing of its corresponding verbal label (Baumgaertner et al., 2007). Moreover, Tremblay et al. (2012) showed that a transient suppression of excitability within left ventral premotor cortex induced by repetitive TMS can result in a disruption of semantic priming during the processing of hand action sentences.

**Figure 1:**
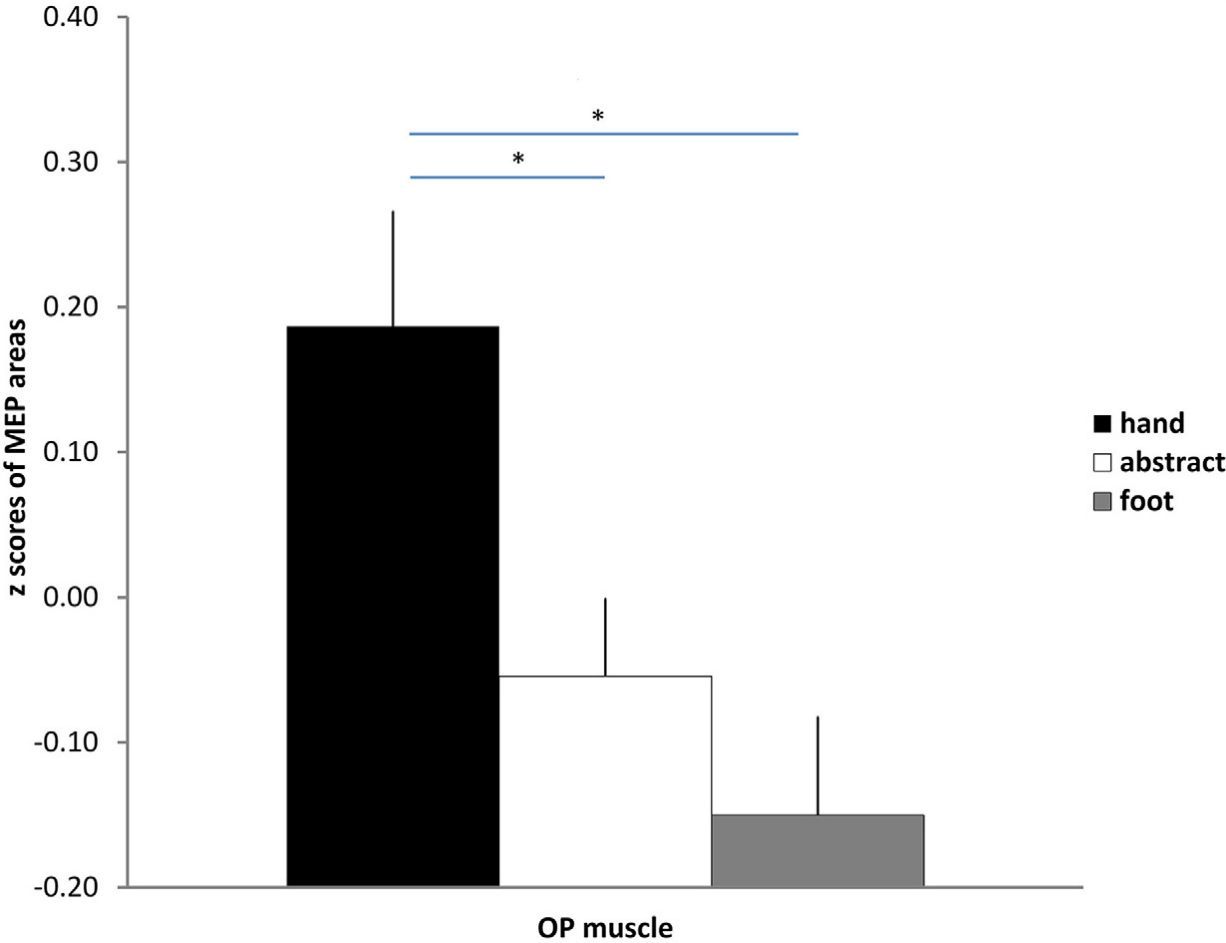
**Mean z-score values for MEPs recorded from the OP muscle in response to hand, abstract and foot sentences**. Bars are SE, ^*^indicates *p* < 0.05.

In contrast, a TMS study by Papeo et al. (2009) showed that MEPs recorded from hand muscles were modulated only at a late interval (500 ms) after stimulus onset and not at shorter intervals (170 and 350 ms). In particular, MEPs were enhanced for the semantic task and reduced for the syllabic one only at a post-conceptual stage of language processing. The authors thus claimed against an automatic activation of the motor system and argued that the observed facilitation for the semantic task was due to a post-comprehension motor imagery that in turn activated M1, as a result of action verb understanding. However, by looking carefully at the data see Figures 1, 3 of the original paper of Papeo et al. (2009), it appears that at early stimulation times MEPs recorded during hand-related verbs presentation are reduced as compared to non-hand action verbs. Even though the authors interpreted their data as a lack of modulation of the motor system during language processing (which in their opinion should have been in the direction of facilitation), these data do not seem at odds with those of Buccino et al. (2005) as far as they may be read as suggesting a modulation of the motor system that occurs earlier (170 ms) than that claimed by the authors. In the same study, at a later stage of linguistic processing (500 ms) the authors showed an increase of MEPs amplitude and faster RTs when processing hand action related verbs as compared to non-hand action related verbs. The authors interpreted this facilitation effect as the mere result of upstream cognitive processing (Mahon and Caramazza, 2008).

Altogether, the literature reviewed so far supports the notion of a fine-tuned modulation of the motor system with an early recruitment that in TMS studies may manifest itself as an interference effect and a delayed recruitment manifesting itself as a facilitation effect and likely expressing the results of a post-conceptual language processing.

Within this theoretical framework, we believe that a replication of the study by Buccino et al. (2005) is useful for several reasons. First, we aimed to enlarge the sample size and thus replicate the findings with higher statistical power. Additionally, we proposed an improvement of EMG recording and a refinement of the experimental methods aimed at making data clearer and more straightforward. Specifically, we recorded MEPs from just one muscle for each effector (*opponens pollicis* for hand and *tibialis anterior* for foot/leg) and carried out both the behavioral and TMS experiment recruiting only male participants, to rule out potential gender differences in linguistic or motor processes.

We predicted an interference effect occurring at an early time interval post-stimulus presentation. Replicating this effect would then support the conclusion that the motor system recruitment occurs at early stages of verb processing, thus supporting its causative role for this cognitive function (possibly even in the absence of an explicit task).

Moreover, the findings of this replication may serve as the basis for further experiments.

## Methods

The present replication consists of two experiments, a neurophysiological study using single-pulse TMS and a behavioral one, with two separate samples of participants. All methods, including estimated samples, data analysis, exclusion criteria, and expected results were submitted before data collection. The experiments were performed according to the registered methods.

### Experiment 1: TMS study

The aim of this experiment was to assess whether listening to action-related sentences modulates the activity of M1. Namely, whether the amplitude of MEPs induced in hand and foot/leg muscles by the stimulation of the corresponding motor areas is modulated by passive listening to different types of sentences. As stated in the original study, the aim of stimulating both hand and foot representation and recording MEPs from the correspondent muscles is to test the specific modulation by action sentences on the related bodily effector.

To this aim, hand- and foot-related sentences were presented, as well as sentences with abstract content as controls.

### Participants

With the software G^*^Power (Version 3.1.6, University of Duesseldorf) we calculated the effect size for each experiment based on the original ANOVA tables and then computed the required sample size in order to obtain a statistical power of at least 0.95.

For the TMS experiment, we planned two separate ANOVAs for hand and foot motor cortex stimulation, considering “sentence” as a within-participants variable with three levels (hand-related, foot-related and abstract sentences). For this reason, we first calculated the effect size for the main effect of “sentence” from the original study. Since G^*^Power allows the use of Partial eta squared as direct input of effect size, we applied the formula η^2^_*p*_ = *SSeffect*/*SSeffect* + *SSerror*. The result is η^2^_*p*_ = 0.455 for the hand and η^2^_*p*_ = 0.415 for the foot. Subsequently, we used these values as input for the G^*^power sample estimation (under the option “effect size estimation as in Cohen”), setting the alpha level at 0.05, group = 1 and measurement = 3. The required sample size was equal to 21 for the hand and to 24 for the foot TMS.

Since both hand and foot motor representations were to be tested in the same participants and taking into account the particularly strict TMS guidelines for recruitment and testing, we decided to take the value of 21 as the expected sample size for the whole TMS experiment. Please note that this is more than 2.5 times more than the original sample in Buccino et al. (2005).

All participants were right-handed native Italian speakers, aged 18–40, with no history of neurological disorders. Handedness was evaluated by means of a standard Edinburgh questionnaire (Oldfield, 1971) and participants were recruited at the Universities of Potsdam and Berlin, and through public advertisement. As a general criterion, only participants reporting to speak and write in Italian at least three times a week were included in the sample. In addition, only male participants were tested, as in the original study the authors reported that only male students were tested due to the difficulty in finding an appropriate and stable hotspot for the foot/leg motor representation in female participants (Giovanni Buccino, personal communication). All participants were screened for possible contraindications and gave their informed consent to the TMS procedure, as required by the University of Potsdam and its Ethics Committee.

### Materials and procedure

The experiment took place in a sound-attenuated room and consisted of two sessions performed in two separate days (for every participant, same time of day and, whenever possible, day of the week of two consecutive weeks).

Participants were sitting on a comfortable armchair, with their elbow flexed at 90°, with their hands prone and their leg flexed at 90° in a relaxed position. The head of the participants was lying on a headrest in order to maintain a comfortable and stable position. In each experimental session, either the hand motor area or the foot motor area of the left hemisphere was stimulated by means of single-pulse TMS delivered by a Magstim Rapid2 stimulator (Magstim Company, Whitland, UK) and a standard 70 mm figure-of-eight coil placed on the skull with a medio-lateral orientation (handle pointing backwords). MEPs were recorded from the right *opponens pollicis* (OP), when the hand motor area was stimulated, and from the right *tibialis anterior* muscle (TA) when the foot motor area was stimulated.

As compared to the original study, the use of only one muscle for each effector constitutes a methodological improvement in full agreement with the replication attempt. Indeed, the original study showed that hand muscles were equally affected by the sentence type, with no main effect or interaction of the factor “muscle.” This is supported by the fact that both muscles are recruited while performing the presented hand-related actions. On the contrary, the analysis on MEPs recorded from leg muscles showed that only the TA was significantly affected by sentence type. The authors of the original study suggest that this effect depends on the prevalent involvement of TA in the actual performing of presented leg-foot actions. The data from the original study support the choice of testing only one muscle for each effector, which is also in line with other TMS studies (e.g., Papeo et al., 2009) recording only one muscle for the hand. Finally, the quality of the data should increase by allowing the choice of an optimal hotspot for each muscle.

Participants wore a swimming cap with a grid of 1-cm resolution drawn on it. Following the international 10–20 EEG system, the coordinate origin was fixed at the Vertex. Moving the coil on the grid by 1-cm steps, either the hand or, possibly, the foot motor area were localized at the beginning of each session. For every participant, stimulus intensity was adjusted in order to determine the resting motor threshold for each of the recorded muscles. During each experimental session, stimulus intensity was set at 120% of the measured threshold.

During the two experimental sessions, participants were asked to carefully listen to different acoustic stimuli consisting of hand- or foot-related or abstract content sentences. The sentences were the same for the two sessions, while the sessions differed in the stimulated sector of the motor cortex and the corresponding recorded muscle (hand/foot). The order of the two experimental sessions was counterbalanced across participants.

Acoustic stimuli were delivered at a fixed intensity (80 dB) by means of two loudspeakers connected to a computer and the software Eprime (Psychology Software Tools, Inc.) that was also in charge of triggering the TMS stimulation. While the digital recordings of the sentences (in Italian, see Table 1) used in the original study were made available by the original authors, the original timings indexing the onset of the second syllable for each verb were no longer available. For this reason, the exact timing of the second syllable (corresponding to the TMS pulse timing) was re-detected on the stimulus spectrogram (under the supervision of Giovanni Buccino) by means of the software SASLAB (Avisoft) and employing the same procedure followed in the original study.

**Table 1:** **Hand-, foot- and abstract content-related sentences used in the two experiments**.

**Hand-related sentences**		**Foot-related sentences**		**Abstract content sentences**	
**Italian**	**Translation**	**Italian**	**Translation**	**Italian**	**Translation**
**TMS AND BEHAVIORAL**					
Cuciva la gonna	He sewed the skirt	Calciava la porta (x2)	He kicked the door	Amava la moglie	He loved his wife
Girava la chiave	He turned the key	Calciava la palla (x2)	He kicked the ball	Amava la patria	He loved his country
Lavava i vetri	He cleaned the glasses	Calciava la sedia (x2)	He kicked the chair	Godeva la vista	He enjoyed the sight
Prendeva la tazza	He took the cup	Marciava sul posto	He marched on the place	Gradiva la mela (x2)	He liked the apple
Scriveva il tema	He wrote the essay	Pestava la coda (x3)	He stepped on the tail	Odiava la guerra (x2)	He hated the war
Sfilava il filo	He slipped off the wire	Pestava l'erba	He stepped on the grass	Pativa il caldo	He suffered from the heat
Sfogliava il libro	He leafed through the book	Pestava le foglie	He trod on the leaves	Scordava la data	He forgot the date
Spalmava la crema	He smeared the cream	Saltava la corda	He jumped the rope	Scordava il nome	He forgot the name
Spezzava il pane	He broke the bread	Saltava il fosso	He jumped the ditch	Serbava la fede	He kept the faith
Stringeva la mano	He shook the hand	Saltava il muro	He jumped the wall	Soffriva il freddo	He suffered from the cold
suonava il piano	He played the piano			Temeva la legge	He feared the law
Svitava il tappo	He unscrewed the cap			Temeva la pena	He feared the penalty
Tagliava la carne	He cut the meat			Vinceva la gara	He won the competition
Tagliava la stoffa	He cut the cloth				
Timbrava la busta	He stamped the envelope				
**BEHAVIORAL**					
Apriva la porta	He opened the door	Ballava il tip tap	He danced the tiptap	Bramava la gloria	He longed for the glory
Firmava il contratto	He signed the contract	Ballava il tango	He danced the tango	Covava l'odio	He harbored hatred
Graffiava il viso	He scratched her face	Correva la gara	He ran the race	Credeva il vero	He believed the truth
Lanciava la palla	He threw the ball	Danzava alla Scala	He danced at the Scala	Intuiva il fine	He sensed the aim
Potava il ramo	He was pruning the branch	Saliva le scale	He climbed the stairs	Negava la verità	He denied the truth
Spremeva il frutto	He squeezed the fruit			Provava il vero	He demonstrated the truth
Strappava il foglio	He ripped the paper			Sognava il mare	He dreamed the sea
				Studiava la storia	He studied history

As in the original study, some foot and abstract content sentences were repeated more than once to have an equal number of sentences for all the three types of stimuli.

Two main types of sentences were used: action-related sentences (hand- and foot-related) and abstract sentences (as controls), see Table 1. All action-related sentences express a concrete content, describing an action performed on a concrete object (e.g., “cuciva la gonna” translation “he/she sewed the skirt”) with either the hand or the foot/leg as body effector. All abstract content sentences describe an abstract action performed on an adequate object (e.g., “amava la patria” translation “he loved his land”). All verbs are composed by three syllables and are conjugated in the third person of the past tense. The past tense in Italian is composed by adding the suffix “−va” to the verbal stem, e.g., “cuci”+ “va” for “cuciva” (he/she sewed). Fifteen hand-related and fifteen foot-related action sentences constituted the final set of stimuli, together with 15 abstract-related sentences (all sentences matched for familiarity in the previous study). As in the original paradigm, sentences were presented in blocks. Each block contained only one type of sentence (hand-related, foot-related or abstract), with the order of blocks counterbalanced across participants.

During the acoustic presentation of sentences, single-pulse stimulation was delivered in correspondence of the second syllable of the verb (e.g., “cuci” for “cuciva”) by means of an external PC trigger. This timing was chosen in order to allow an early stimulation when the verb stem is likely to have been just understood by participants. On average, the timing of the second syllable (pulse delivery) occurred 603 ms (*SD* = 76) after the beginning of the sentence, according to the length of the verb. Each experimental session comprised 45 sentences, with a 65 s interval between two consecutive pulses. Continuous EMG recording by means of surface Ag-AgCl electrodes allowed the recording of MEPs in correspondence of the presented sentences. EMG traces were band-pass filtered (20–1000 Hz), digitized (sampling frequency 2000 Hz) and stored on a computer for off-line analysis. After rectification, the area underlying MEPs was calculated for each trial and used for successive statistical analysis. The pre-TMS electromyographic activity, starting 100 ms before TMS, was also acquired in all trials in order to check for any possible difference between conditions.

### Data analysis

MEP areas of all subjects were normalized (z scores) separately for the two sessions (hand motor area or foot/leg motor area stimulation). The data were analyzed with two separate repeated measures analysis of variance (ANOVA), one for the effector hand and one for the foot, with “sentence” (hand-, foot-, and abstract-related sentences) as within-participants variable. Pairwise comparisons with the Newman–Keuls method were conducted whenever appropriate. The significance level was set at 0.05 and η^2^_*p*_ was reported as a measure of effect size.

### Expected results

According to the data from the original study, we expected to find a similar pattern for both hand and foot effectors, showing reduced MEPs when processing sentences involving the same effector (hand- and foot-related, respectively) whose motor representation was magnetically stimulated. In particular, we expected to find a significant main effect of “sentence” as follows:

- for the effector hand (MEPs from the OP muscle), reduced MEP amplitude when processing hand verbs as compared to the foot ones;
- for the effector foot (MEPs from the TA muscle), reduced MEP amplitude when processing foot verbs as compared to the hand ones.

Accordingly, we did not expect any modulation related to abstract-content sentences, as they are not expected to activate any motor-related information.

Possible alternative results might concern two aspects: the direction of the effect (increase, i.e., facilitation vs. decrease, i.e., interference) and the presence of the effect in only one of the bodily effectors, regardless of the direction. The latter considers the fact that previous studies already showed the effect on hand-related verbs processing and hand responses as more stable and reliable. The former takes into account data from previous studies (such as Papeo et al., 2009) showing facilitation in case of action sentence/stimulated bodily effector congruency (e.g., hand-related action verbs and hand motor cortex stimulation), with increased MEPs. A result in this direction would not question the assumption that MEPs are affected by congruency between sentence and stimulated effector at early stages of language processing, since timing is not manipulated in our replication. On the other hand, a similar outcome would support the idea that a facilitation effect accompanies motor resonance during verb processing similarly to what happens during action observation, thus questioning the hypothesis that when the motor system is engaged in both a linguistic and a motor task (or stimulation of the motor cortex, in absence of an overt motor task), participants pay a cost.

### Experiment 2: behavioral study

Since the TMS study focused on an implicit task (no explicit request of deep semantic processing), a second behavioral study used a go-no go paradigm in order to test whether RTs are affected by the semantic processing involved in distinguishing between action- and abstract-related types of sentences.

### Participants

A new sample of volunteers took part in the second experiment, none of them having participated to the first experiment. Participants were selected at the Magna Graecia University of Catanzaro, Italy, following the same criteria described for Experiment 1.

As in Experiment 1, in order to estimate the required sample size, we first calculated the effect size for “sentence” and “effector” interaction from the original ANOVA table. Since G^*^Power allows the use of η^2^_*p*_ as direct input of effect size, we applied the formula η^2^_*p*_ = *SSeffect*/*SSeffect* + *SSerror*. In this case we have a mixed (2 × 2, within/between design) and thus used the G^*^Power option of “ANOVA repeated measures within-between interaction” (effect size estimation as in Cohen). By entering the η^2^_*p*_ = 0.348, alpha = 0.05, groups = 2 and measurements = 2 we obtained a sample size estimation equal to 28 participants (14 for each group).

The original study tested an equal number of male and female participants. However, since in the TMS study we planned to recruit only male participants, we tested only male participants also in this second experiment. We believe this is a minor change in the original design, which nevertheless makes the TMS and the behavioral experiments more comparable and rules out potential gender effects.

### Materials and procedure

The experiment was carried out in a sound-attenuated room, dimly illuminated by a halogen lamp directed toward the ceiling (lighting conditions as described in the original study), while participants were sitting in front of a computer screen at a viewing distance of 50 cm.

Participants were randomly divided into two groups. The first group used the right hand as the response effector, while the second group used the right foot.

As in Experiment 1, stimuli were delivered in acoustic form. Participants again listened to three different types of sentences: hand-action-related sentences, foot-action-related sentences, and abstract-content-related sentences. Some sentences were the same as in Experiment 1, some others were new (see Table 1), but with the same syntactic structure. Some sentences were presented twice or three times during the experiment. In addition, thirty catch trials (see below) were presented, for a total of 150 trials for the entire experiment and in a single session.

Participants were instructed to carefully listen to each sentence and respond either with their hand or with their foot when the sentences describe a concrete action (hand- and foot-related sentences), and refrain from responding when the sentences describe an abstract action. Sentences were delivered by means of two loudspeakers, both located at the same distance from participants' ears (about 50 cm) and driven by a PC. According to the original design, and differently from Experiment 1, sentences were not presented in blocks, but randomly presented within the same block by means of the software Eprime, as in Experiment 1.

Each trial began with the appearance of a white circle at the center of the computer screen. Soon after, a sentence was acoustically presented. In correspondence with the second syllable of the verb—exactly as for the TMS timing in study 1—the circle became green. This occurred between 500 and 700 ms, depending on the length of the verb. The green spot acted as go-signal for participants. In 20% of trials the go-signal was delivered later, in coincidence with the second syllable of the object noun or at the end of the sentence. These trials avoided habituation to the go timing (catch trials) and were not analyzed further.

For hand responses, they pressed the 0-key of the numerical keypad positioned on the right side of a keyboard in front of the computer screen. During the experiment, participants' right index finger was positioned over the key and, when required, the response was given by pressing the key. For foot responses, participants responded by means of a quadrangle-shaped pedal (6 cm side). During the experiment, participants' right foot rested on the pedal and participants pressed the pedal whenever required by the experimental task.

Trials with RTs faster than 130 ms (anticipations) or slower than 1000 ms (missing responses) were considered errors and discarded. A maximum of 10% of all trials was allowed for errors (anticipations, missing responses, wrong semantics) and any participant exceeding this limit was excluded from further analysis.

### Data analysis

For each participant, median values were calculated for correct RTs in relation to each type of sentence. These values were submitted to an ANOVA with “sentence” (hand or foot action) as a within-subject variable, and “effector” (hand or foot) as between-subject variable. Pairwise comparisons with the Newman–Keuls method were conducted whenever appropriate. The significance level was set at 0.05 and η^2^_*p*_ were reported as measure of effect size.

### Expected results

According to the original study, we expected to find a significant interaction between “sentence” and “effector.” Namely, we expected to find the same pattern in both groups as follows:

- for the effector hand, significantly slower RTs when responding to hand verbs as compared to the foot ones;
- for the effector foot, significantly slower RTs when responding to foot verbs as compared to the hand ones.

Finding an interference effect would support the interpretation according to which this effect stems out when the motor system is activated for both understanding action sentences and planning motor responses, thus paying a cost in terms of RTs.

Finding an effect in the opposite direction (facilitation instead of interference) would not question the fact that RTs (similarly to MEPs) are affected by congruency between sentence and stimulated effector at early stages of language processing. On the other hand, and similarly to what stated for the TMS experiment, such an outcome would question the hypothesis that when the motor system is engaged in both a linguistic and a motor task behavioral performance is worse and participants pay a cost in terms of slower motor responses. This possible finding would then be in line with previous studies claiming that motor resonance is accompanied by a facilitation effect.

Similarly to MEPs, we have to consider that replicating the effect for only one of the two effectors would specifically put into question the choice of motor responses that do not fully match how much a specific effector is engaged in the target sentences/actions.

## Results

### Experiment 1: TMS study

Twenty-one right-handed male participants were included in the analysis of the hand MEPs but only seven of these were also included in the analysis of foot MEPs. The remaining 14 participants could not be included because of the difficulty to evoke stable MEPs from the TA muscle at rest, remaining within the safety and comfort guidelines for TMS use. These difficulties were expected (see Methods section) but major technical modifications, such as for instance the use of a different coil in the foot session, were not pursued in order to stay within the constraints of an exact replication.

Five additional participants were tested but are not included into the final analysis: one completed the experiment but was excluded because of the poor quality of the EMG signal, four did not complete the experiment and decided voluntarily to leave/skip the second session.

The repeated measures ANOVA on hand MEPs showed a significant effect of “sentence”: [*F*_(2, 40)_ = 4.39, *p* < 0.05, η^2^_*p*_ = 0.18]. *Post-hoc* Newman–Keuls tests showed that MEPs in response to hand-related sentences were significantly larger than the ones for foot-related (*p* = 0.02) or abstract sentences (*p* = 0.046), as depicted in Figure 1. On the contrary, foot-related and abstract sentences did not significantly differ (*p* = 0.42).

The repeated measures ANOVA on foot MEPs showed no significant effect of “sentence”: [*F*_(2, 12)_ = 0.32, *p* = 0.72, η^2^_*p*_ = 0.05] and MEPs in response to foot-related sentences were on average only slightly smaller (mean z-score = −0.120) than the hand-related ones (mean z-score = 0.111).

### Experiment 2: behavioral study

Forty-four right-handed male participants took part in the behavioral experiment, divided into two groups of twenty-two participants each.

Eleven additional participants were not included into the analysis because their global error rate (anticipations, missing responses, wrong semantics) exceeded 10%.

**Figure 2:**
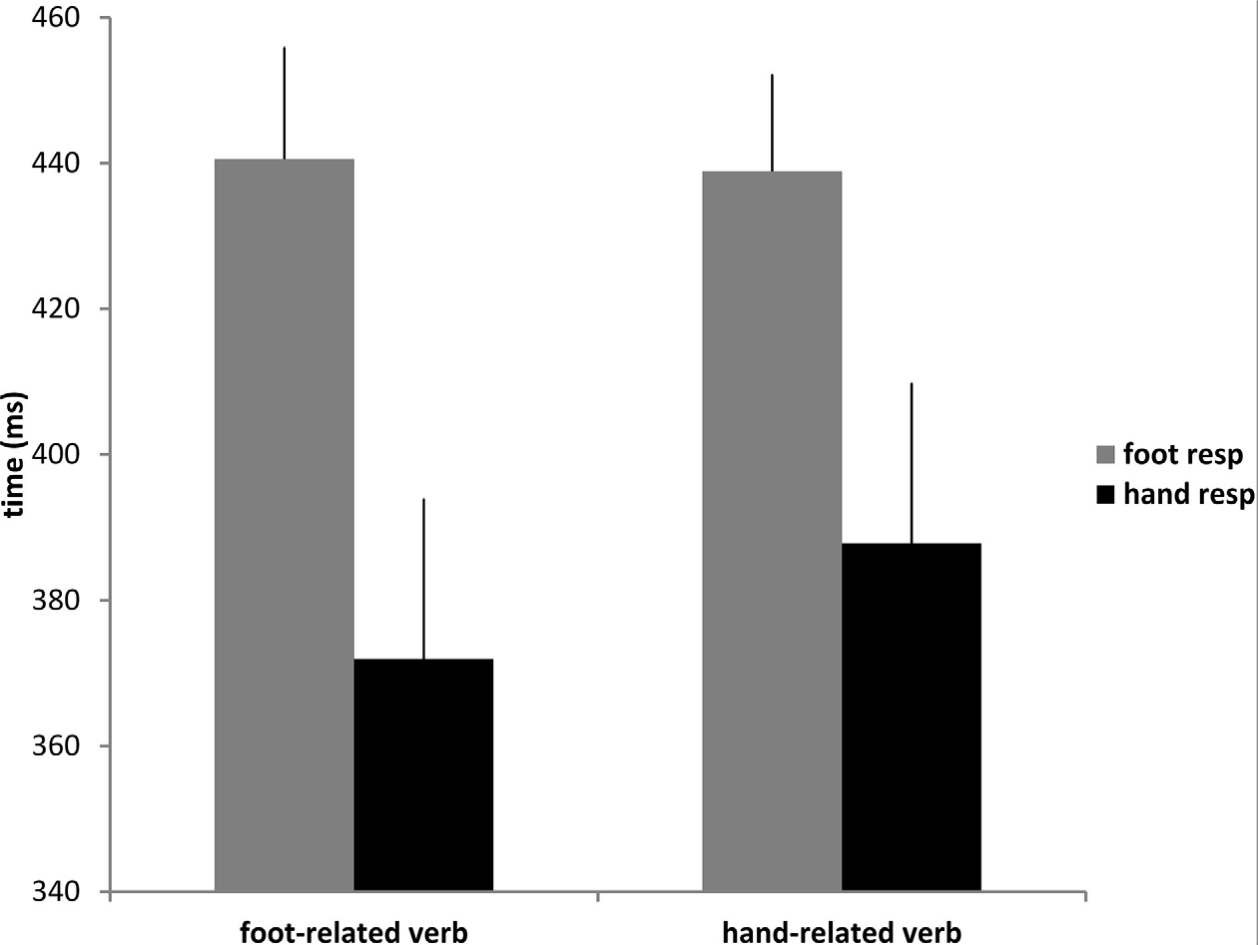
**Mean reaction times for foot and hand responses to foot and hand-related sentences**. Bars are SE.

The repeated measures ANOVA revealed a main effect of “effector” [*F*_(1, 42)_ = 4.99, *p* < 0.05, η^2^_*p*_ = 0.1], indicating that responses given by the foot (440 ms) were slower than responses given by the hand (380 ms). The main effect “sentence” and the interaction “sentence” x “effector” did not reach significance [*F*_(1, 42)_ = 0.77, *p* = 0.38, η^2^_*p*_ = 0.18 and *F*_(1, 42)_ = 1.18, *p* = 0.28, η^2^_*p*_ = 0.02, respectively]. As shown in Figure 2, on average the hand responses to hand-related sentences were slower than those to foot-related sentences (388 vs. 372 ms), and also foot responses to foot-related verbs were slightly slower (441 vs. 439 ms).

## Discussion

### Experiment 1: TMS study

The discussion of this experiment only considers the data from the hand MEPs. Unfortunately, we were not able to replicate the foot part of the experiment with an adequate number of participants and power and consequently we cannot reach any conclusion.

As expected for hand stimulation, our data showed an early modulation of MEPs, according to the congruency between the linguistically described actions and the stimulated effector. This early modulation is consistent with the data from the original study and supports the idea of an early involvement of the motor system in language processing.

However, the direction of the effect is reversed with respect to Buccino et al. (2005), where an interference effect was shown. Namely, hand MEPs increased in response to hand-related action sentences, as compared to foot-related and abstract ones. Nevertheless, these findings are coherent with previous data on action observation and action-language processing. Classically, since the seminal work by Fadiga et al. (1995), experiments on action observation have shown a robust increase of MEPs in case of congruency between the stimulated effector and the observed action (for a review see Avenanti et al., 2013). Our results support the claim that similar brain mechanisms are at play both when actions are observed and when they are only linguistically experienced.

Crucially, our data show this facilitation occurs at early stages of auditory language processing and in absence of any overt task: these are central issues in the debate regarding how peculiar the involvement of the motor system in language processing might be. Papeo et al. (2009) have criticized the original study by Buccino et al. (2005) and claimed that the late facilitation observed in their study is connected to post-conceptual processing and strategies of motor imagery. Our experiment, designed as a direct replication of Buccino et al. (2005) with increased sample size, suggest that motor facilitation during language processing is not only a late effect related to post-conceptual stages. However, since the timing of the stimulation was not manipulated, we cannot show what would have happened with earlier or later TMS timings. In addition, the definition of the exact timing for TMS stimulation in the case of auditory stimuli certainly requires further investigation as, for instance, the direct comparison of written and auditory stimuli.

In the present study, MEPs modulation was clearly shown in absence of any overt semantic or motor task. Since the instructions were as close as possible to the original ones, participants were just requested to carefully listen to the presented sentences. While this seems to support a certain degree of automaticity of this process, the absence of an overt task does not allow us to rule out strategies of motor imagery applied by participants. However, we believe that the possibility that the present MEPs modulation reflects motor imagery is unlikely because the TMS pulse was delivered soon after the acoustically presented verb became meaningful (i.e., close to the isolation point of the verb). Another relevant point regards the type of sentences we used: as they were all in third person perspective (see Table 1), our results support the idea that the facilitation is quite robust and resists to a rather “external” perspective (in contrast to Papeo et al., 2011). However, since most of the data showing a certain flexibility of motor resonance as a function of the linguistic perspective come from behavioral studies (for a review see Beveridge and Pickering, 2013), we cannot rule out the possibility that what is evident as facilitation at the neurophysiological level can turn in a reduced facilitation or interference when different perspectives are compared and a behavioral response is requested.

### Experiment 2: behavioral study

Despite more than doubling the number of participants, we were unable to replicate the most significant findings in the behavioral experiment. In particular, we were not able to get significant results in the crucial “sentence” × “effector” interaction. The only significant effect, namely that the hand responses were faster than the foot ones, might give us a hint regarding a possible confound given by comparing different types of motor responses in a within-between design. A more straightforward design should take these motor differences into account.

It is worth noting, however, that a qualitative look at the data shows that the hand responses to hand verbs tended to be modulated in the sense of an interference, although only slightly. Therefore, a possible explanation applying at least to hand responses is that the effect size computed from the original data of Buccino et al. (2005) was overestimated. This is likely due to the small sample size of the original study.

As far as foot responses are concerned, a possible explanation for the failure in showing any modulation of these responses according to the bodily effector expressed by the verb comes from the fact that the type of foot action used to give the motor response (i.e., a pedal press) did not fully match the foot actions described in the target sentences. Future experiments should probably aim at carefully looking for a closer similarity between the muscular pattern involved in the action expressed by a sentence and the muscular pattern involved in the motor response, as it is already the case for specific target muscles in TMS experiments.

A final consideration concerns the timing of the second syllable of the verbs. This timing is indeed crucial because it represents the timing of the magnetic pulse delivery for the TMS experiment and the timing of the go signal for the behavioral one. Since the timings of the original study were no longer available and had to be newly determined manually, we cannot exclude that our estimation of these values might not exactly fit the original ones. However, we believe this possibility is actually remote since particular attention was paid when inspecting the acoustic spectra of the stimuli and it is unlikely to have affected the outcome of our experiments. However, we believe that future studies may benefit from taking seriously into account the need for more explicit and precise rules for the determination of the TMS timing (or go-signal) in case of auditory stimuli, in order to improve replicability and comparison across studies.

## Conclusions

The present study attempted to replicate a well-known, but controversial study in the field of embodied language processing, by increasing its power and sample size.

While the TMS experiment produced an early modulation of hand MEPs during action-language processing, the direction of the effect was reversed. As discussed, further studies are needed in order to investigate how specific the observed facilitation is, and under which conditions (if any) it can possibly turn into interference. In addition, this experiment gave us fundamental information regarding the estimation of the effect size. The same holds for the behavioral experiment, although in this case no significant interaction was shown. In addition, our replication gave important methodological insights, especially for future TMS studies aiming at a final clarification regarding the true nature and timing of the involvement of the motor system in language processing.

### Conflict of interest statement

The authors declare that the research was conducted in the absence of any commercial or financial relationships that could be construed as a potential conflict of interest.
